# High-Resolution Drone Detection Based on Background Difference and SAG-YOLOv5s

**DOI:** 10.3390/s22155825

**Published:** 2022-08-04

**Authors:** Yaowen Lv, Zhiqing Ai, Manfei Chen, Xuanrui Gong, Yuxuan Wang, Zhenghai Lu

**Affiliations:** College of Optoelectronic Engineering, Changchun University of Science and Technology, Changchun 130022, China

**Keywords:** object detection, background difference, high-resolution image, drone, small target

## Abstract

To solve the problem of low accuracy and slow speed of drone detection in high-resolution images with fixed cameras, we propose a detection method combining background difference and lightweight network SAG-YOLOv5s. First, background difference is used to extract potential drone targets in high-resolution images, eliminating most of the background to reduce computational overhead. Secondly, the Ghost module and SimAM attention mechanism are introduced on the basis of YOLOv5s to reduce the total number of model parameters and improve feature extraction, and *α*-DIoU loss is used to replace the original DIoU loss to improve the accuracy of bounding box regression. Finally, to verify the effectiveness of our method, a high-resolution drone dataset is made based on the public data set. Experimental results show that the detection accuracy of the proposed method reaches 97.6%, 24.3 percentage points higher than that of YOLOv5s, and the detection speed in 4K video reaches 13.2 FPS, which meets the actual demand and is significantly better than similar algorithms. It achieves a good balance between detection accuracy and detection speed and provides a method benchmark for high-resolution drone detection under a fixed camera.

## 1. Introduction

With the increasingly widespread use of consumer unmanned aerial vehicles (UAVs), UAV jamming has brought great pressure to air defense security. The key to fighting against malicious UAV systems is effective detection of small and fast-moving UAVs or drones. With the popularity of high-resolution shooting equipment, there is an increasing demand for processing high-resolution data. Especially in the vision detection and tracking of small targets moving long distances, more detailed information of small targets can be obtained. Therefore, high-resolution equipment is more and more widely used in the detection of drones. Drones are characterized by their small size, high-flying altitude, and fast-movement speed, which brings great challenges to drone detection under high-resolution images [1].

In early studies, most methods detected small drones through radar systems, radio detection, and other technologies [2], but there are certain limitations due to cost, complexity, and coverage. Due to the strong feature extraction capability of deep neural network, methods based on CNN are increasingly applied to drone-detection tasks. In [3], to solve the problem of insufficient drone data collection, a data set of artificial drones and birds was made by subtracting the background of the target and combining with other real pictures, and YOLOv2 was used to achieve accurate detection of drones and birds. In [4], the segmentation network, U-Net, was first used to extract areas of interest (ROI), and ResNet was then used to classify the targets in ROI, finally realizing the detection of drones. For drone detection under fixed cameras, a recurrent correlation network (RCN) based on convolutional neural network, recurrent neural network, and correlation filtering was proposed in [1], which extracts the motion features of tiny flying objects in 4K video by joint detection and tracking to improve detection performance. In [5], the authors proposed a two-stage method. First, background subtraction was used to extract potential targets, and then the CaffeNet network was used to classify potential targets and filter out interference targets, such as birds. This method filters out the background through complex background modeling and obtains the location coordinates of the target. However, when the foreground target is incorrectly extracted or not completely extracted in the background-modeling stage, the target recognition and location will be biased. Therefore, this paper uses the simple background difference method to extract candidate regions in the image, and then uses the target-detection network to classify and precisely locate the targets in the candidate regions.

**Figure 1:**
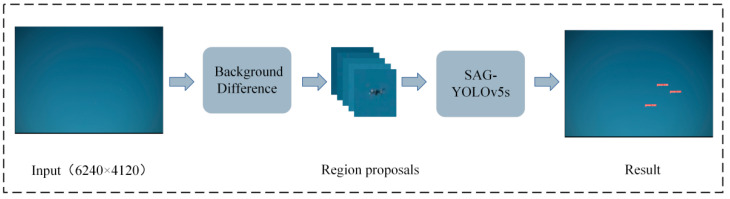
The steps in this paper to detect high-resolution drones with fixed cameras.

Object detection is one of the basic tasks of computer vision, which includes target classification and target location. Some algorithms with excellent performance, such as YOLOv5, Faster-RCNN [6], and CenterNet [7], have achieved excellent performance in many detection tasks. Among them, YOLOv5 is a one-stage algorithm, which is widely used due to its high-detection accuracy and fast-inference speed. But for small drones with targets in high-resolution images, the detection effect is poor. In this paper, we improved YOLOv5 to obtain SAG-YOLOv5s, a lightweight detection network with high-detection accuracy and combined it with background difference for the high-resolution drone detection under fixed cameras. In SAG-YOLOv5s, we introduced a lightweight Ghost module [8] to reduce the total number of model parameters and improve the detection speed. Then, to improve the network’s attention to the drone targets and suppress the complex background, the SimAM [9] attention mechanism was integrated. In addition, the DIoU loss [10] in YOLOv5s was replaced by the *α*-DIoU loss [11] to improve the bounding box regression accuracy and make the drone target localization in high-resolution images more accurate. As shown in Figure 1, the high-resolution drone detection is divided into two steps. First, possible targets are extracted as candidate regions through background difference. Then, all the candidate regions are fed into the lightweight detection network, SAG-YOLOv5s, to perform efficient detection.

## 2. High-Resolution Drone Detection

### 2.1. Extracting Potential Targets through Background Difference

Background difference is a commonly used video-target-detection algorithm, which has important applications in intelligent monitoring and other fields [12]. As background difference has advantages of high-segmentation accuracy and fast-operation speed, it can be used to extract potential targets in high-resolution video images under fixed cameras.

Before the detection, we calculate the pixel level gray median of image sequences as the background model, B(x,y), and then, the video images are subtracted from the background model to obtain the difference image. As follows:

(1) $$D(x,y)=\left|I_{t}(x,y)-B(x,y)\right|$$

where, D(x,y) represents the difference image, $I_{t}(x,y)$ represents the current video image sequences. Binary operations are performed on the difference images to obtain the thresholded image, R(x,y), and its operation expression is shown in Formula (2).

(2) $$R(x,y)=\{\begin{array}{c} 1,D(x,y)>T\\ 0,D(x,y)\leq T \end{array}$$

where, T represents a fixed pixel threshold used to distinguish the foreground and background. The higher the threshold, the higher the processing efficiency will be, but the missed-detection rate will increase. The lower the threshold, the slower the processing speed will be, and the false-detection rate will increase. According to the complexity of the background and through experiments, we set a reasonable pixel threshold, T, to effectively extract potential drone targets and improve the processing efficiency.

**Figure 2:**
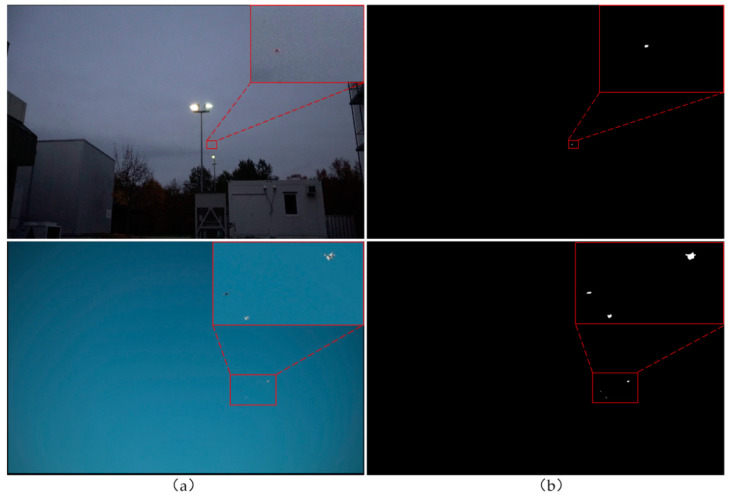
Extraction of potential targets: (**a**) original images; (**b**) results obtained by background difference.

When the thresholded image is obtained, the morphological operations are carried. Since the target is small and the image resolution is high, the closing operation with kernel 18 is carried out first and then perform an opening operation with a kernel of 3. The closing operation is to dilate and then erode the thresholded image, and the opening operation is to erode and then dilate the thresholded image. In this way, noise interference can be removed, and some holes or gaps can be filled on the premise of retaining tiny targets to obtain foreground targets. Figure 2 shows the original images and the results obtained by background difference.

**Figure 3:**
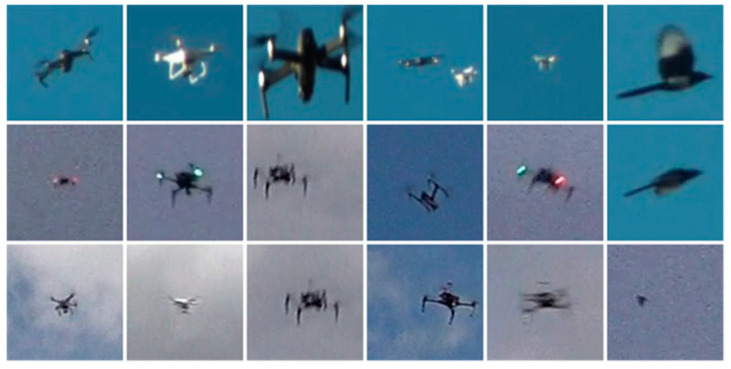
Examples of candidate areas that may contain interference targets, such as birds.

Since small drones occupy very small pixels in high-resolution original images, and drones at long distances are usually blurred, it is crucial to make full use of the context information of the targets for drone detection. Therefore, the foreground target is enlarged to a uniform size in the original image as a candidate region, and the size of the candidate region is controlled within a reasonable range to perform efficient detection. Some examples of candidate regions extracted by background difference are shown in Figure 3.

### 2.2. SAG-Yolov5s, a Lightweight Detection Network

**Figure 4:**
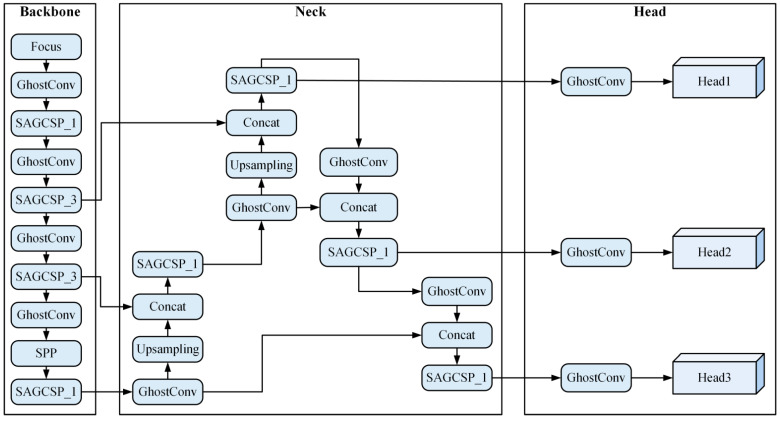
SAG-YOLOv5s object detection network.

YOLOv5 is an efficient target detection algorithm developed by the Ultralytics team [13]. Compared with most other one stage detectors, it has higher detection accuracy and faster inference speed. This paper proposes an improved lightweight-object-detection network, SAG-YOLOv5s, based on YOLOv5s, as shown in Figure 4. The algorithm introduces the Ghost module and SimAM attention mechanism based on YOLOv5s, which improves the detection speed while retaining the detection accuracy.

#### 2.2.1. The Ghost Module

The Ghost module is a lightweight structure. Since the feature maps output by ordinary convolution layer usually contain a lot of redundancy, and some of them may be similar to each other, the Ghost module splits the ordinary convolutions into two parts. The first part is a certain number of ordinary convolutions and generates inherent feature maps through convolution operations, and the second part generates more feature maps through a series of simple linear operations. These two feature maps are then stacked to maintain the feature dimension of the output.

**Figure 5:**
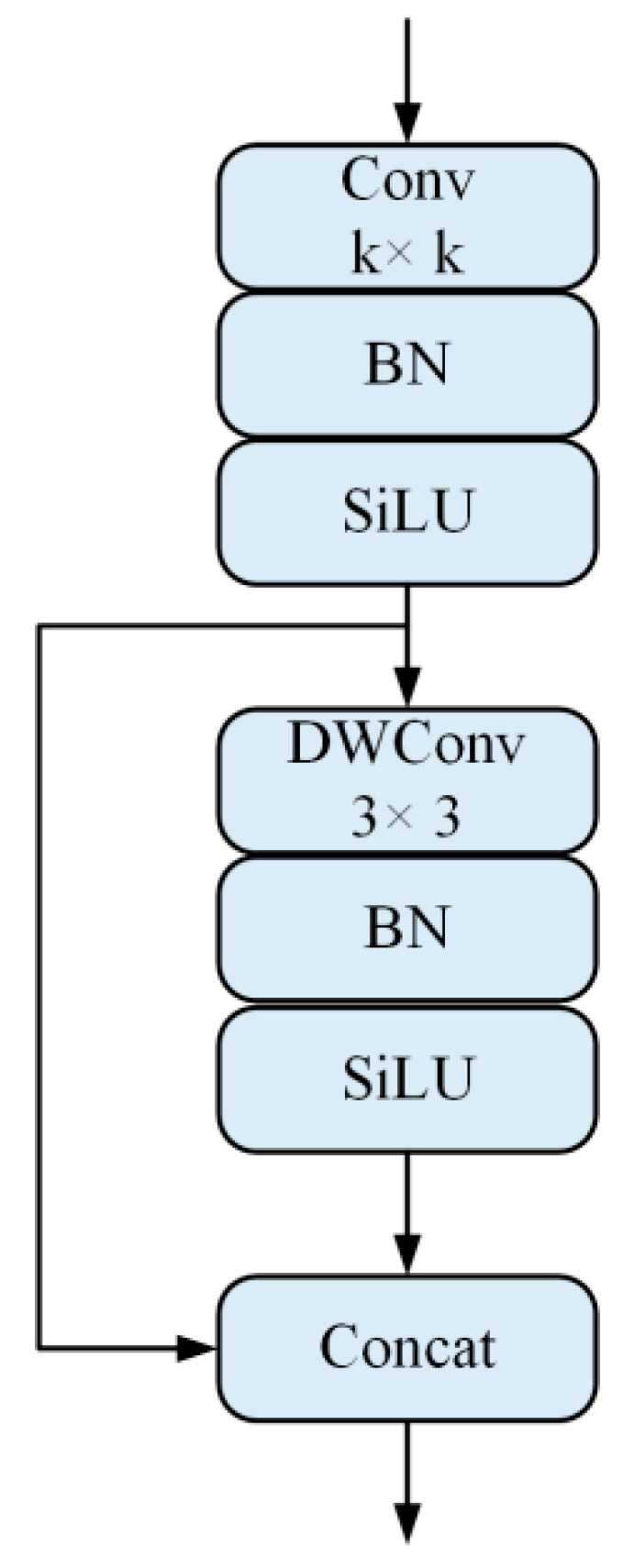
The GhostConv structure.

We use GhostConv to represent the Ghost module introduced into YOLOv5s, and its structure is shown in Figure 5. It can be seen that in the convolution operation of the first part, it is treated with batch normalization (BN) and the (Sigmoid Linear Unit) SiLU activation function is introduced. The main function of the BN layer is to make the input and output of the different network layers in the same distribution to speed up the convergence of the model. SiLU is improved from the Sigmoid activation function to prevent the gradient from disappearing during model training. In the second part, depthwise separable convolution (DWConv) is used for simple linear operations, which are BN processed and SiLU activation function introduced to accommodate the stacking of the two-part feature maps. Compared with ordinary convolution, the Ghost module greatly reduces the computational complexity and the total number of model parameters without changing the size of the output feature maps.

#### 2.2.2. YOLOv5s

YOLOv5s is mainly composed of four parts: input, backbone network Darknet-53 [14], neck network PANet [15], and output. CBS and CSP_n are the basic structures of YOLOv5s, where CBS represents the basic convolution module, which has undergone a BN operation and introduced a SiLU activation function to prevent the gradient from disappearing. The CSP_n structure mainly includes two branches. The first branch is composed of n Bottleneck modules in series, and the second branch is the CBS convolution block. Then, the two branches are stacked. This structure increases the network depth and improves the feature-extraction ability.

The input terminal mainly performs operations, such as data enhancement and adaptive anchor box. The backbone network is mainly composed of 2 CSP_1 and 2 CSP_3 structures for feature extraction. The neck network mainly contains four CSP_1 structures and achieves feature fusion of different levels through multiple up-sampling and down-sampling, realizing multi-scale output, and improving the detection ability of small targets. Additionally, the output head finally achieves accurate detection of the target through bounding box regression and NMS post-processing.

#### 2.2.3. SimAM Attention Module

In many computer-vision tasks, attention modules, such as SE [16] and CBAM [17], are commonly used to improve the model’s attention to the target so that the network can learn more useful information. Different from the existing channel-attention mechanisms or spatial-attention mechanisms, SimAM does not simply connect the channel- and spatial-attention mechanisms in series or parallel, but it is a 3D attention module that combines two attention mechanisms to work together. Inspired by the attention mechanism of the brain, this module designs an energy function to explore the importance of each neuron and derives 3D attention weights for feature maps without additional parameters.

In neuroscience, the active neurons usually suppress the surrounding neurons, a phenomenon known as spatial suppression. We should assign higher importance to neurons with significant spatial inhibition. Therefore, the energy function of SimAM is defined as:

(3) $$e_{t}\left(w_{t},b_{t},y,x_{i}\right)=\frac{1}{M-1}\sum_{i=1}^{M-1}{\left(-1-\left(w_{t}x_{i}+b_{t}\right)\right)}^{2}+{\left(1-\left(w_{t}t+b_{t}\right)\right)}^{2}+\lambda w_{t}^{2}$$

where, t and $x_{i}$ are the target neuron and other neurons in the single channel of input feature, $X\in {\mathbb{R}}^{C\times H\times W}$, *i* is the index of spatial dimension, $\begin{array}{l} M=H\times W \end{array}$ is the number of neurons in a single channel, $\left(w_{t}x_{i}+b_{t}\right)$ and $\left(w_{t}t+b_{t}\right)$ are the linear transformations of $x_{i}$ and t. The minimum energy formula can be obtained by calculating the closed-form solution of $w_{t}$ and $b_{t}$, and the mean and variance of all neurons in the channel.

(4) $$e_{t}^{*}=\frac{4\left({\hat{\sigma }}^{2}+\lambda \right)}{{\left(t-\hat{\mu }\right)}^{2}+2{\hat{\sigma }}^{2}+2\lambda }$$

where $\hat{\mu }=\frac{1}{M}\sum_{i=1}^{M}x_{i}$, ${\hat{\sigma }}^{2}=\frac{1}{M}\sum_{i=1}^{M}{\left(x_{i}-\hat{\mu }\right)}^{2}$. According to the above formula, the lower the energy value, the greater the difference between neuron t and other neurons, and the more important the t neuron is. The SimAM module is finally optimized as:

(5) $$\tilde{X}=sigmoid\left(\frac{1}{E}\right)⊙X$$

where, E is the sum of $e_{t}^{*}$ in all channel and spatial dimensions. The Sigmoid function is used to restrict values too large in E, and it does not affect the relative importance of each neuron. In this paper, the SimAM attention mechanism is embedded in the YOLOv5s network structure, which enables better focus of the drone targets in the feature extraction process without introducing additional parameters.

#### 2.2.4. SAG-YOLOv5s

**Figure 6:**
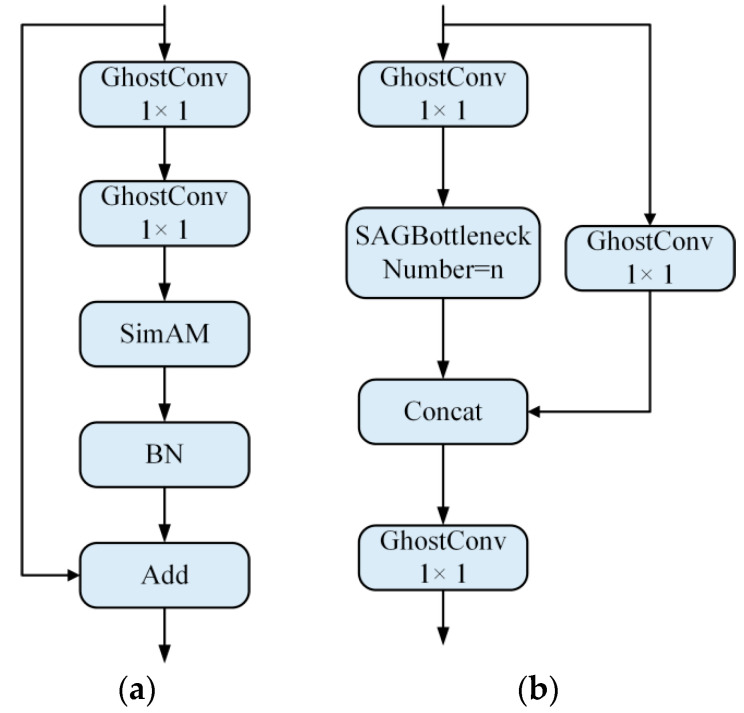
Basic structure of SAG-YOLOv5s: (**a**) SAGBottleneck structure; (**b**) SAGCSP_n structure.

This paper proposes a lightweight object detection network, SAG-YOLOv5s, that integrates the Ghost module and the attention mechanism, which makes the following improvements to YOLOv5s:

1. The CBS basic convolution block is replaced with Ghostconv. Ordinary convolution brings numerous parameters, and the introduction of the Ghost module can greatly reduce the total number of model parameters. According to the Ghost module, for input $X\in R^{c\times h\times w}$ to obtain the n=m×s dimension feature maps, the normal convolution is used to generate the inherent feature maps of the m-dimension, and then, the linear transformations of the inherent feature maps of the m-dimension are performed to generate the m×(s−1) dimension feature maps. Finally, the two parts of the feature maps are stacked to obtain the results. Therefore, the number of common convolution parameters is about s times that of the Ghost module. In this paper, it is assumed that the number of output channels are $c_{2}$, the dimensions of the inherent feature graph are $c_{2}/2$, and the BN term and bias term are simplified. Therefore, the acceleration ratio is about 2, and the number of parameters of GhostConv is only about half of CBS.
2. The SimAM attention mechanism is introduced, and SAGBottleneck is designed as the basic structure of SAG-YOLOv5s. To better suppress the complex background and focus on drone targets during feature extraction, the SimAM attention module and the Ghost module are integrated and embedded into the Bottleneck structure of YOLOv5s. The SAGBottleneck structure is shown in Figure 6a. This structure is similar to the residual structure, mainly including two 1 × 1 Ghostconv modules and a skip connection. The first Ghostconv is used to reduce the number of input channels, thereby reducing the feature dimension. The SimAM attention mechanism is embedded in the second Ghostconv module to improve feature extraction and increase the feature dimensions to keep consistent with the input and finally add the input and output of the left and right parts through skip connections. In the second Ghostconv, the SimAM attention module is fused before the BN operation to adapt to the output, and the SiLU activation function is removed. This is because after the SiLU activation function, the distribution of the input data of the current layer and the next layer are different, which will reduce the model convergence speed during training. In addition, the SAGBottleneck is integrated into the CSP_n structure to obtain the SAGCSP_n structure, as shown in Figure 6b, which consists of 3 and 1 × 1 Ghostconv and n SAGBottlenecks.

Due to the effective fusion of the Ghost module and the SimAM attention mechanism, the feature extraction ability of the network is strengthened, and the amount of parameters is greatly reduced so that SAG-YOLOv5s can improve the detection speed of high-resolution drones while maintaining the detection accuracy.

### 2.3. α-DIoU Loss for Bounding Box Regression

Bounding box regression is a fundamental task in many advanced detectors. The anchor-based detector obtains the predicted bounding boxes of the target by regressing offsets between the ground-truth bounding boxes and their preset anchor boxes. Intersection over Union (IoU) loss [18] is a classic bounding box regression loss function that calculates the localization loss between the resulting bounding boxes and the ground-truth bounding boxes.

To improve the localization accuracy of drone targets and increase the robustness of the proposed algorithm on small data sets, *α*-DIoU loss is used instead of DIoU loss in YOLOv5s. DIoU loss is an improvement on the GIoU loss [19], and compared to the GIoU loss, DIoU loss can directly minimize the distance between the two target bounding boxes, and the convergence speed has been greatly improved. The DIoU loss expression is:

(6) $$L_{\mathrm{DIoU}}=1-\mathrm{IoU}+\frac{\rho^{2}(b,b^{\mathrm{gt}})}{c^{2}}$$

where, b and $b^{\mathrm{gt}}$ represent the central points of the predicted bounding boxes and the real box, respectively. ρ stands for Euclidean distance, and c represents the diagonal length of the smallest enclosing rectangle that can cover both the prediction box and the ground-truth bounding boxes.

*α*-DIoU loss is a unified power generalization of DIoU loss by power parameter, *α*, which can be expressed as:

(7) $$L_{\text{α-DIoU}}=1-{\mathrm{IoU}}^{\alpha }+\frac{\rho^{2\alpha }(b,b^{\mathrm{gt}})}{c^{2\alpha }}$$

The boundary box loss function contains a power IoU term and an additional power regularization term. By adjusting the power parameter, *α*, the *α*-DIoU loss can help improve bounding box regression accuracy by adaptively up-weighting the loss and gradient of high IoU objects. The loss function can make the detector detect more true positive targets and less false positive targets, thus improving the final detection accuracy. In this paper, by adjusting the value of *α*, the accuracy of the boundary box regression is effectively improved without increasing parameters; thus, the drone targets localization is more accurate, and the target detection performance is improved.

## 3. Experiment and Result Analysis

### 3.1. Dataset

Deep neural networks usually require a large amount of real-world data for training. Therefore, to verify the effectiveness of the algorithm in this paper, a high-resolution drone detection dataset is produced based on the public dataset, Drone-vs-Bird [20,21].

**Figure 7:**
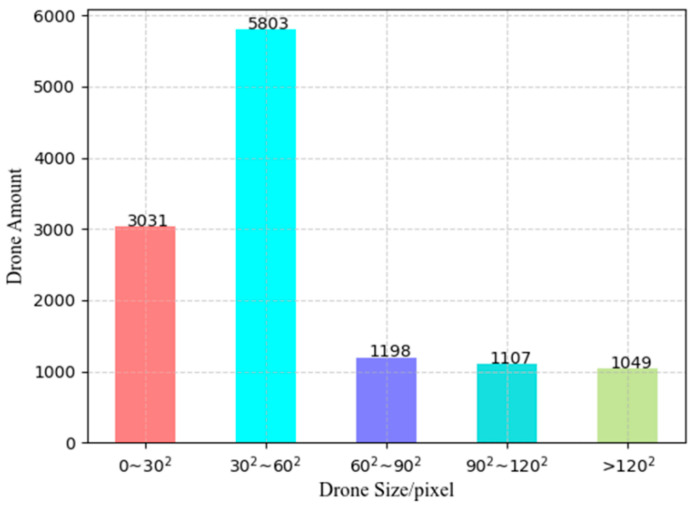
Target size distribution in the high-resolution drone dataset.

We selected several 4K videos in this public dataset with a total of 9410 pictures of 3840 × 2160 pixels and collected several high-resolution video segments containing drones, birds, or other interfering targets on the spot under a fixed camera. We annotated 1876 images (6240 × 4120 pixels) from field-collected videos and mixed their 4K video image sequences together to obtain a high-resolution drone dataset of 11,286 images. The dataset has a total of 12,188 drone targets with an average of 1.08 targets per image. Figure 7 is the size distribution diagram of the objects in the dataset. There are many extremely tiny objects (less than 30 × 30 pixels), and nearly half of the objects are distributed in size between 30 × 30 pixels and 60 × 60 pixels. Most objects have a very small pixel ratio in the original image. In the experiments of this paper, 90% of the data is used for training and validation, and 10% of the data is used as the test set to evaluate the model.

### 3.2. Experimental Results and Evaluation

The experiments in this paper are carried out on the Ubuntu18.04 system, the CPU is Intel (R) Core i5-10400F @ 2.90 GHz, the GPU is Nvidia GeForce RTX 2070 SUPER, the GPU acceleration software is CUDA10.2 and CuDNN7.6.5, and the deep-learning framework used is Pytorch1.8.1. The main evaluation indexes selected in the experiment are accuracy (*P*), recall (*R*), and average accuracy mean (mAP). The formula is as follows:

(8) $$P=\frac{TP}{TP+FP}\times 100\%$$

(9) $$R=\frac{TP}{TP+FN}\times 100\%$$

(10) $$\mathrm{AP}=\int_{0}^{1}P(r)dr$$

where, TP (true positives) represents the number of correctly predicted positive examples, FP (false positives) represents the number of falsely predicted positive examples, and FN (false negatives) represents the number of incorrectly predicted negative examples. AP is the average precision, which is expressed as the integral of the precision rate to the recall rate. When detecting a class, the size of mAP is its AP value.

In the training phase, the data set is preprocessed, and all the candidate regions extracted from the background difference are used for the training of the algorithm in this paper. In the inference stage, the high-resolution original image is directly used as the input. First, the candidate region is extracted through the background difference, and then, SAG-YOLOv5s classifies and precisely locates the target in the candidate region and finally obtains the detection result through NMS. Since most drone targets are extremely tiny relative to the original image, a lower value is used as the IoU threshold in the NMS stage to filter out redundant overlapping boxes.

**Figure 8:**
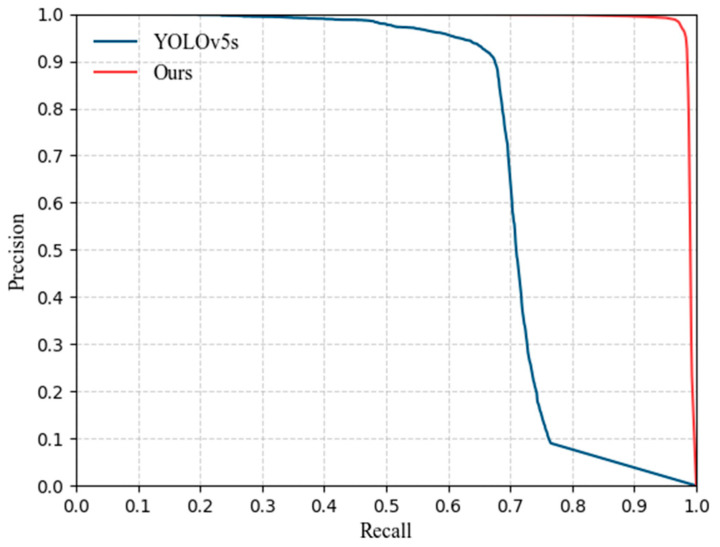
*PR* curves of the proposed algorithm and YOLOv5s.

Figure 8 shows the *PR* curves comparison between the proposed algorithm and the original YOLOv5s. It can be seen that compared with YOLOv5s, the *PR* curve obtained by our method covers a larger area with the X and Y axes indicating that it has better performance in the detection of high-resolution drones.

To further verify the effectiveness of our method, ablation experiments are performed. We mainly evaluate the detection performance and model complexity. The experimental results are shown in Table 1, where BD represents the background difference, and FPS represents the number of frames per second processed by the algorithm. Parameters and floating-point operations (FLOPs) are used to measure the complexity of the neural network model. The smaller the value, the lower the model complexity. The following conclusions can be drawn from Table 1:

1. From the data in the first and second rows, it can be seen that after using the background difference, the mAP value increased from 73.3% to 97.0%, an increase of 23.7%, this is because the original YOLOv5s directly reduces the high-resolution image to a fixed size and then performs feature extraction during detection, which is easy to cause small targets to be lost, and it is difficult to extract effective features, resulting in a poor detection effect.
2. It can be seen from the data in the third and second rows, as well as the data in the fifth and fourth rows that after introducing the SimAM attention mechanism, mAP is increased by 0.5% and 1.8%, respectively, indicating that SimAM can pay more attention to drone targets in the process of feature extraction and show better results on lightweight networks.
3. Comparing our SAG-YOLOv5s network and YOLOv5s network, the amount of floating-point operations is reduced from 16.4 GFLOPS to 8.8 GFLOPS, which shows that our network model has lower model complexity. After combining the background difference and SAG-YOLOv5s, the detection speed when the candidate areas are 416 × 416 reaches 13.2 FPS, which is 4.2 FPS higher than before the introduction of the Ghost module;
4. Comparing the data in the sixth row and the fifth row, it can be seen that reducing the size of the candidate region from 416 × 416 to 96 × 96 can improve the detection speed, but it will lead to a slight decrease in mAP. This is mainly because of the 96 × 96 input image, which reduces the detection accuracy of larger size drones. In specific applications, actual adjustments can be made according to the distance between the fixed camera and the monitored drone.

In addition, our method is compared with some other drone detection methods. Table 2 shows the experimental results of CenterNet, YOLT [22], and ours. All results are obtained on the same test set, and the bold font in the table indicates the best results. Among them, YOLT is a commonly used high-resolution image detection method, which uses three steps of cutting, detection, and merging to detect small targets in high-resolution images. YOLT-YOLOv5s means replacing the detector in YOLT with advanced YOLOv5s. According to the results in Table 2, CenterNet has a poor detection effect on high-resolution drones because the small targets are lost after the original images are reduced and after multiple down sampling. Although YOLT and YOLT-YOLOv5s have high-detection accuracy for high-resolution drones, the detection speed is slow due to the large number of sub-images after cutting the high-resolution images, which takes up a lot of memory, while our method achieves 97.6% mAP on the test set and is faster. It shows a good-detection ability for high-resolution drones and achieves a good balance between detection accuracy and detection speed.

### 3.3. Results Visualization

**Figure 9:**
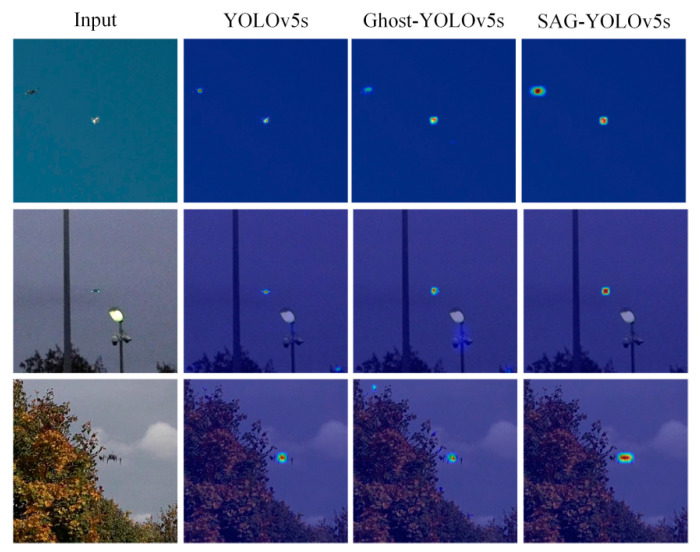
Visual heatmaps of different networks.

Finally, to verify the effectiveness of introducing the SimAM attention module, the visualization heatmaps of the YOLOv5s, Ghost-YOLOv5s, and SAG-YOLOv5s networks after feature extraction of the targets in the candidate areas are generated according to the Grad-CAM [23] method, as shown in Figure 9.

Compared with the original algorithm YOLOv5s, after combining the Ghost module, there is a small amount of noise interference on the heatmaps of the Ghost-YOLOv5s. During the feature extraction process, it is easily interfered by similar objects in the surrounding environment, and the attention to the targets is not enough, resulting in a decrease in the detection effect. After the SimAM attention mechanism is effectively integrated with the Ghost module, the color of the target areas on the SAG-YOLOv5s heatmaps become significantly darker, which effectively strengthens the target feature extraction, suppresses the complex background area, and makes the network more focused on the drone targets. According to the visual analysis, it can be concluded that the SAG-YOLOv5s proposed in this paper can well reduce the interference information and improve the target detection accuracy.

**Figure 10:**
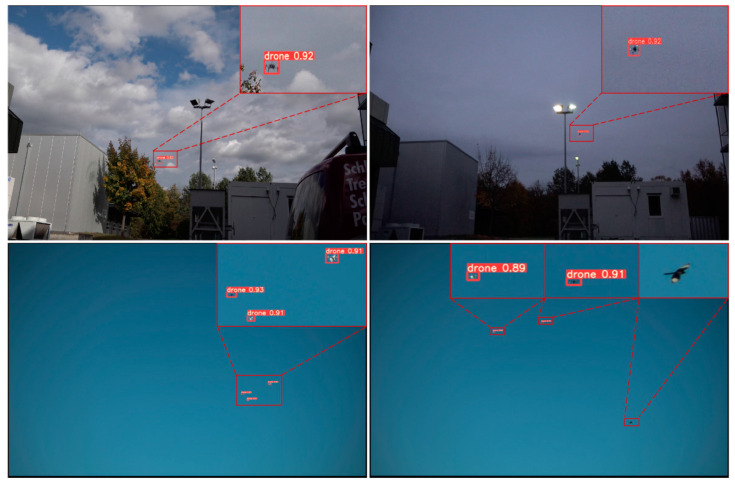
The high-resolution drone detection results obtained by the method in this paper.

Figure 10 shows the drone detection results of the proposed method in high-resolution images. It can be seen that under the fixed camera, our method can well detect tiny drones and filter out interference targets, such as birds, which has practical application value.

## 4. Conclusions

In this paper, we proposed an efficient method for detecting small drones in high-resolution images. Aiming at the problem of high-image resolution and small target, a two-stage method is proposed to detect drone targets under a fixed camera. In the first stage, potential drone targets are extracted by background difference, and numerous background areas are excluded, which greatly improves the detection efficiency of high-resolution images. In the second stage, the lightweight detection network SAG-YOLOv5s, which integrates the Ghost module and the SimAM attention mechanism, is used to efficiently and accurately detect targets in candidate areas. Finally, using *α*-DIoU loss improves the object bounding box regression accuracy. We made a high-resolution drone dataset for experiments based on the existing dataset. The experimental results show that compared with the original YOLOv5s and some other drone detection algorithms, our method has obvious advantages in high-resolution drone detection under fixed cameras, which improves the detection speed while maintaining the detection accuracy.

## Figures and Tables

**Table 1:** Ablation experiments.

Method	mAP@0.5 (%)	Parameters (10^6^)	FLOPs (10^9^)	FPS	Input Size (pixels)
YOLOv5s	73.3	7.06	16.4	21.0	416 × 416
BD + YOLOv5s	97.0	7.06	16.4	9.2	416 × 416
BD + YOLOv5s + SimAM	97.5	7.06	16.4	9.0	416 × 416
BD + Ghost + YOLOv5s	95.8	3.90	8.8	13.4	416 × 416
BD + SAG-YOLOv5s	97.6	3.90	8.8	13.2	416 × 416
BD + SAG-YOLOv5s	97.2	3.90	8.8	15.0	96 × 96

**Table 2:** Experimental results of various algorithms on the test set.

Method	Precision (%)	Recall (%)	mAP@0.5 (%)	FPS
CenterNet	87.2	69.5	69.8	10.5
YOLT	92.9	88.0	91.1	4.6
YOLT-YOLOV5s	95.3	92.8	95.2	6.0
Ours	**97.3**	**95.5**	**97.6**	**13.2**

## Data Availability

Not applicable.
